# Inflammatory Mechanisms Associated with Skeletal Muscle Sequelae after Stroke: Role of Physical Exercise

**DOI:** 10.1155/2016/3957958

**Published:** 2016-08-28

**Authors:** Hélio José Coelho Junior, Bruno Bavaresco Gambassi, Tiego Aparecido Diniz, Isabela Maia da Cruz Fernandes, Érico Chagas Caperuto, Marco Carlos Uchida, Fabio Santos Lira, Bruno Rodrigues

**Affiliations:** ^1^Faculty of Physical Education, University of Campinas (UNICAMP), 13083-851 Campinas, SP, Brazil; ^2^Exercise and Immunometabolism Research Group, Department of Physical Education, São Paulo State University (UNESP), 19060-900 Presidente Prudente, SP, Brazil; ^3^Human Movement Laboratory, São Judas Tadeu University (USJT), 03166-000 São Paulo, SP, Brazil

## Abstract

Inflammatory markers are increased systematically and locally (e.g., skeletal muscle) in stroke patients. Besides being associated with cardiovascular risk factors, proinflammatory cytokines seem to play a key role in muscle atrophy by regulating the pathways involved in this condition. As such, they may cause severe decrease in muscle strength and power, as well as impairment in cardiorespiratory fitness. On the other hand, physical exercise (PE) has been widely suggested as a powerful tool for treating stroke patients, since PE is able to regenerate, even if partially, physical and cognitive functions. However, the mechanisms underlying the beneficial effects of physical exercise in poststroke patients remain poorly understood. Thus, in this study we analyze the candidate mechanisms associated with muscle atrophy in stroke patients, as well as the modulatory effect of inflammation in this condition. Later, we suggest the two strongest anti-inflammatory candidate mechanisms, myokines and the cholinergic anti-inflammatory pathway, which may be activated by physical exercise and may contribute to a decrease in proinflammatory markers of poststroke patients.

## 1. Introduction

Chronic stroke is the second leading cause of death and the third cause of disability worldwide. Moreover, the overall incidence of stroke is increasing exponentially. From 1990 to 2010, the number of deaths and disabilities related with stroke rose by 26% and 19%, respectively, regardless of the age group [1, 2]. Indeed, every year approximately 795,000 people experience a new or recurrent chronic stroke, and every four minutes someone dies from stroke in the United States [3].

Furthermore, it has been shown that stroke prevalence is greater in low-income countries when compared to developed countries. A systematic review comprising 56 epidemiological studies carried out between 1970 and 2008 showed that in ten countries of low and middle income the incidence and premature mortality due to chronic stroke more than doubled, reaching 5.6% increase annually [4].

Muscle atrophy in the paretic and nonparetic limbs is a phenotypic change caused by different factors (e.g., hemiparesis and immobilization) observed in poststroke patients [5]. Since this atrophy is associated with decrease in muscle strength and power, cardiovascular fitness, and mobility, some researchers have suggested that poststroke patients show stroke-related sarcopenia, similar to the muscle weakness found in elderly people [6, 7]. Regardless of the molecular pathway responsible for eliciting this phenomenon, several studies have indicated that increased inflammatory markers may be a trigger factor for this condition [8, 9]. In fact, some findings from research have pointed that proinflammatory cytokines (e.g., TNF-*α* and IL-6) may activate the molecular pathways involved muscle atrophy (e.g., ubiquitin proteasome system).

On the other hand, physical exercise has been widely suggested as a beneficial tool for rehabilitation of stroke patients, since it may be able to counterregulate most of the stroke sequelae on the organic system [10]. Indeed, several reviews have suggested that physical exercise may elicit improvement on cognition, upper and lower limb motor function, cardiovascular performance, cardiovascular risk factors (e.g., triglycerides and insulin resistance), fatigue resistance, balance, gait, and mobility [10–12]. Also, physical exercise has been effectively used as an anti-inflammatory therapy in chronic diseases [13–16].

Unfortunately, data about the mechanisms underlying the beneficial effects of physical exercise on poststroke patients are limited. However, several experiments have already demonstrated that physical exercise may decrease the inflammatory state through myokines and the cholinergic anti-inflammatory pathway; as such, we may hypothesize that both mechanisms may also be present in stroke patients. In this sense, the present study attempted to demonstrate the potential role of inflammation on muscle atrophy, regardless of specific molecular pathways in poststroke patients. We also assessed the compensatory role of myokines and the cholinergic anti-inflammatory pathway in counterbalancing this phenomenon and consequently improving prognosis.

## 2. Functional and Skeletal Muscle Complications Associated with Stroke

Stroke complications are numerous and variable depending on the site of impairment and the degree of obstruction of the blood vessels involved. Generally, stroke complications may be characterized as morphological (e.g., decrease in muscle mass and increase in muscle mass fat infiltration), physical (e.g., hemiparesis, spasticity, rigidity, balance and coordination changes, tremors, deficit in gross and fine motor skills, and sensory changes), psychoaffective (e.g., depressive disorders, anxiety, and aggressiveness), and cognitive (memory, attention, and concentration issues, language disorders and executive functions, difficulty in action planning, and perceptual deficit) [6, 7, 17].

Regarding physical alterations, hemiparesis or muscle weakness contralateral to brain lesion, which is characterized by weakness/palsy in one of the sagittal body sides and central nervous system injury, is the most frequent disability found in poststroke patients, affecting around 50% of the patients [6, 18–21].

Hemiparesis is closely associated with muscular abnormalities, which, in turn, cause impairment in muscle functionality [6, 18]. Indeed, studies have found poststroke atrophy in the contralateral limb, whereas results on the ipsilateral limb have demonstrating maintaining or even trend to increase in the muscle mass [20, 22, 23]. Moreover, evidences demonstrate nondifferences between the muscle volume of the quadriceps from the ipsilateral limb of poststroke patients in comparison with the limb of control group matched by age and sex [23]. In the following 3 months after a stroke, patients show small differences in muscle mass content between the contralateral and ipsilateral limb with women and men displaying 1.88% and 3.74% lower muscle mass in the paretic limb and nonparetic limb, respectively [22]. However, with the progression of the condition, differences between sagittal sides may amount to 24% in 6 months [20, 23].

In line with these findings, a systematic review, which evaluated 14 studies, undertaken in three different continents (i.e., America, Europe, and Asia) and involved 450 poststroke patients (i.e., 53.5–75 years), has shown that the contralateral limb displayed 4.5% atrophy when compared to the ipsilateral limb, with a high magnitude of atrophy observed in the midthigh (14.5%) [5].

Decrease in muscle mass due hospitalization is associated with poor prognosis, including length of stay in intensive care unit in different population [24, 25]. In poststroke patients, decrease in muscle mass few days after the phenomenon is associated with impairment in the capacity to walk again [26]. Furthermore, evidence suggests that this association is dependent on the affected limb, as demonstrated by Prado-Medeiros et al. [23], who found a moderate but significantly negative association between paretic limb atrophy (but not in ipsilateral limb) and the capacity to generate strength and power of the muscle knee flexors and extensors [23].

The paretic limb shows lower isometric and isotonic strength than ipsilateral limb [20, 27]; in turn, both show lower strength than nonaffected healthy control limbs [27]. These physical characteristics involved in the capacity to generate strength and power in poststroke patients deserve attention, since low strength is positively associated with low scores in tests that mimic the activities of daily life (ADL), such as six-minute walk test (6MWT), stair climbing (i.e., normal and fast pace), gait speed (i.e., normal and fast pace), moving from a sitting to a standing position, flexing the affected knee, and scales, which evaluate muscle function (i.e., Rivermead motor assessment), in poststroke patients [23, 28, 29]. Moreover, patients who suffered stroke seem to spend more energy to perform these daily tasks [29].

Due these stroke-induced alterations on muscle mass, physical function, and functionality, some studies have suggested that the term sarcopenia should be employed to describe this phenotype found in poststroke patients [6, 7]. According to the these investigations, this aspect is clinically important since sarcopenia has been found to be a trigger factor to development of syndromes associated with low resilience in elderly people, such as frailty syndrome [30]. However, muscle and functional alterations in poststroke patients seems to occur in different times and magnitudes, unlike the changes observed in elderly people [6, 7, 18, 31].

Muscle fiber shifting helps explain these differences. In older people, predominantly, histological analyses show type II fast-twitch shift toward to type I low-twitch muscle fibers [32, 33]. On the other hand, poststroke patients undergo an inverse phenomenon, whereby type I low-twitch muscle fibers shift forward to type II fast-twitch muscle fibers [6, 18]. These histological changes are inversely associated with gait speed and are likely to be caused by altered neural activation patterns [34]. However, consensus about sarcopenia diagnosis in poststroke patients has not yet been reached, as well as the role it plays in the physical function in this population [6, 7, 19].

## 3. Possible Mechanisms Associated with Muscle Atrophy in Poststroke Patients

Some mechanisms have been suggested to explain muscle atrophy in poststroke patients [6, 7]; however, little is known about them [6, 7]. Muscle atrophy is caused by an imbalance between protein synthesis and breakdown and can be elicited by two phenomena: (a) when the rate of protein breakdown (i.e., proteolysis) exceeds the rate of protein synthesis and (b) when the rate of protein synthesis decreases [35, 36].

The insulin-like growth factor 1 (IGF-1)/Akt/mammalian target of rapamycin (mTOR) pathway (i.e., IGF-1/Akt/mTOR) has been known to be the most important pathway to muscle protein synthesis [36].

IGF-1 is a circulating growth factor synthesized in different tissues, including the skeletal muscle [37, 38]. IGF-1 may act either as a hormone, due to its systemic characteristic, or in an autocrine fashion, as a local growth factor [38]. Activity of IGF-1 on skeletal muscle is a strong stimulus to muscle hypertrophy [38, 39]. Indeed, a transgenic mice model with overexpressing of IGF-1 gene has shown higher muscle mass in fore and hind limbs, due to increase in the muscle cross-sectional area of type II fibers, during adult life and older age, in comparison with the wild type [38].

The activity of IGF-1 is mediated by its binding to tyrosine kinase IGF-1 receptor in the lipid bilayer [36, 40, 41]. IGF-1 recruits insulin receptor substrate (IRS-1), which is also activated by insulin and, consequently, leads to the phosphorylation and activation of phosphatidylinositol 3-kinase (PI3K) [36, 40]. PI3K phosphorylates phosphatidylinositol (4, 5) bisphosphate, synthetizing phosphatidylinositol (3, 4, 5) [36, 39–41]. This process allows the creation of a lipid-binding site to Akt [36, 39–41]. Downstream targets of Akt are the mammalian target of rapamycin (mTOR), which, in turn, phosphorylates and activates p70s6k, also called S6K1, through the essential regulatory subunit eIF3f, ribosomal proteins, which eventually leads to increased ribosomal biogenesis and protein translation [41–44]. Still regarding eIF3f, this regulatory subunit acts as a scaffold, allowing the activation of mTOR downstream targets (i.e., p70^S6K^) [42]. This phenomenon occurs from the binding of mTOR on C-terminal of the eIF3f subunit, phosphorylating and activating p70^S6K^, which is found anchored in the mov34 domain [42]. Evidences indicate that increase of MAFbx in response to food deprivation causes eIF3f loss, leading to marked decrease on phosphorylation of p70^S6K^, without alterations in mTOR activity, indicating the association of mTOR downstream targets [42]. On the other hand, overexpression of eIF3 subunit elicits significantly an increase in P70^S6K^ [42]. It is important to mention that 4E-BP1, a dual activity molecule, can be a key factor in muscle mass regulation, since its molecule can activate eIF3f, after be phosphorylate by mTOR, which leads to its dissociation of eIF3f [45]. However, eIF3f inactivation can occur if 4E-BP1 is dephosphorylated [45].

Moreover, results from experiments* in vitro* and* in vivo* have demonstrated that IGF-1/PI3K/Akt/mTOR pathway may revert muscle proteolysis and muscle myofibrillar breakdown (i.e., actinin), by inhibiting E3 ligases (i.e., MAFbx and MuRF-1), FoxO1—which also activates MAFbx and MuRF-1—, and decrease in the activity of the ubiquitin proteasome system [36, 37, 39, 46].

In an animal model of stroke, atrophied paretic and nonparetic muscles show a downregulation of the IGF-1/PI3K/Akt/mTOR pathway [47]. In poststroke patients, few studies have focused on the association between IGF-1 and muscle atrophy [48]. In the studied performed by Silva-Couto et al. [48], chronic stroke patients with hemiparesis showed lower serum concentrations of IGF-1 and IGFB-3, an IGF-1 transporter, than healthy controls. Concomitantly, muscle atrophy was found in poststroke patients [48]. Other studies have shown a positive correlation between muscle function and IGF-1 serum concentrations, which may indicate a modulation by muscle mass [49]. However, this variable was not measured. Therefore, inferences about the IGF-1 pathway on muscle atrophy in poststroke patients are still sensitive and we must be proceeding with caution.

There is more considerable evidence in the literature for muscle atrophy elicited by the increased rate of protein breakdown than by protein synthesis.

As aforementioned, muscle atrophy is one of the components of the poststroke phenotype [6, 7, 18]. Besides the contribution of hemiparesis to this phenomenon, bed rest time, which occurs from one to three days after the event, immobilization, and decrease in physical activity levels negatively affect muscular homeostasis in poststroke patients, leading to muscle atrophy due to denervation, unloading, and disuse [7, 26, 50].

Although disuse atrophy is probably not regulated by a single mechanism, but a complex one [36], the ATP-dependent ubiquitin proteasome system (UPS) has been widely suggested as the main mechanism responsible for muscle atrophy, since UPS genes were found to be increased in muscular atrophy due to different factors (i.e., denervation, unloading, and disuse) [36, 37, 40, 43]. Ubiquitin is a small peptide that targets the protein, leading to ubiquitin-dependent protein catabolism and forming the core of a much larger protease, the 26S proteasome [40, 43]. The process of ubiquitination, protein labeling and targeting, is dependent on three ubiquitin-enzymes: E1 ubiquitin-activating enzyme, an E2 ubiquitin-conjugating enzyme, and an E3 ubiquitin-ligating enzyme [36, 40]. These enzymes act in a chain reaction fashion creating a polyubiquitination chain, where E1 activates, through an ATP-dependent pathway, and transfers the ubiquitin to E2, which is later replaced by E3 [36, 37, 40, 51], which, in turn, catalyzes the conjugation of the ubiquitin with the target protein [40, 51].

In the skeletal muscle, two muscle-specific E3 enzymes have been studied and were found to be associated with the muscle atrophy phenotype [36, 37, 40]. Muscle ringer finger 1 (MuRF1) and muscle atrophy F-box (MAFbx), also called Atrogin-1, are two E3 ligases widely expressed on muscle mass during muscular atrophy and activated by FoxO family of transcription factors, which remained inactivated by Akt phosphorylation [9, 36, 37, 40]. Besides the activity of MuRF1 and MAFbx on muscle catabolism as an E3 ligase, these genes are associated with the inhibition of the components of muscular anabolism, such as a myogenic regulatory factor and eIF3-f [42, 51].

Both genes seem to be increased in response to different models of muscle atrophy (i.e., denervation and disuse) [43]. In fact, in healthy subjects, for example, MAFbx and MuRF1 expression (i.e., mRNA) has been found to remain increased after the first 10 days of disuse atrophy in lower limb biopsies [52, 53]. Data from experiments in animals deficient in MAFbx and MuRF-1 (MAFbx^−/−^ and MuRF-1^−/−^) genes corroborate evidence about the role of both factors in muscle atrophy, since MAFbx^−/−^ and MuRF-1^−/−^ animals show lower magnitude of loss in muscle mass and fibers during atrophy when compared to the wild type [43].

Although the results in healthy subjects demonstrate a strong association between UPS, particularly MAFbx and MuRF-1 activity, and muscle atrophy, evidence about poststroke patients are limited, and only data from animal studies are available [47, 54].

In the experiment developed by Desgeorges et al. [47], mice undergoing transient focal cerebral ischemia have shown quadriceps, soleus, and tibialis anterior atrophy in the paretic side. Concomitantly, the expression of MuRF-1 and MAFbx did increase in the paretic muscle. Data from Springer et al. [54] corroborate these findings and show increased proteasome activity in the muscle under atrophy after transient focal cerebral ischemia [54].

Together with UPS, autophagy represents the two major proteolytic systems in mammalian cells [55, 56]. Autophagy may be characterized as a homeostatic process which controls the degradation of damaged organelles, toxic proteins, and intracellular pathogens [55, 56]. The extreme activity of the autophagic complex is harmful to muscle mass, since up- and downregulation of autophagic genes leads to muscle wasting [55]. Regarding downregulation, atg7^−/−^ mice, which present autophagy inhibition, show degenerative changes in muscle mass, lower myofiber size (~40%), and muscle strength when compared to the controls, together with increased activity of atrogenes (i.e., MuRF-1 and MAFbx), which can indicate a relation between both factors [55, 56]. Furthermore, during catabolic condition (i.e., fasting and denervation), inhibition of autophagy increases the magnitude of muscle loss [55].

In poststroke patients, autophagy has been associated with muscle atrophy [47, 57]. However, these findings remain controversial. In an animal model of stroke, the expression of genes associated with autophagy complex (i.e., Ulk1, LC3, and cathepsin L) was increased in paretic and nonparetic limbs [47]. However, protein content did not follow the expression of mRNA and remained unaltered. Yet according to some researchers, the lack of changes in autophagy genes does not rule out the association between the autophagic complex and poststroke muscle atrophy, since a delayed increase in proteins cannot be discarded [47]. In humans, data from a number of studies seem to corroborate the findings of Masiero et al. [55]. In fact, patients with chronic spastic hemiplegia and visible muscle atrophy show decreased expression of autophagy genes when compared to healthy older adults [57].

Myostatin has also been suggested as one of the main pathways regulating muscle atrophy in poststroke patients [47, 58]. Myostatin, also known as growth differentiation factor 8 (GDF8), is key muscle protein regulator factor of the transforming growth factor-*β* (TGF-*β*) superfamily of growth and differentiation factors [59, 60]. GDF8 seems to act as a negative factor against muscle mass development from embryogenesis to adult life, impairing muscle synthesis and increasing muscle catabolism [59–61].

Data from observations in animal models knockout to myostatin gene show that the mutant animal displayed from two- to threefold more muscle mass when compared to the control animals throughout life [61–63]. Although this phenomenon is marked by an increased number of muscle fibers (i.e., hyperplasia) during the first years of life, increase in muscle cross-sectional area (i.e., hypertrophy) is predominant in animals undergoing treatment with a myostatin inhibitor (JA16) during adult life [61, 63]. In humans and in cattle breeds, a mutation in the myostatin gene leads to its downregulation, causing abnormal development of muscle mass [64, 65].

Myostatin consists of two terminals: an N-terminal propeptide, which inhibits the activity of myostatin, and a C-terminal, which is the active form of the protein [60]. Before activation, myostatin is secreted and remains in a latent form [60]. Once activated, the activity of myostatin is regulated by binding with the serine/threonine transmembrane receptor [59, 60, 66]. After being activated by myostatin, the activin type II receptor recruits, phosphorylates, and activates the activin type I receptor, which is associated with SMAD proteins [59, 60, 66]. These proteins are one of the mechanisms responsible for the effect of myostatin on muscle cells, since phosphorylated SMAD 2 and SMAD 3 build a complex with SMAD 4, which is then translocated to the nucleus and changes the transcription of target catabolic genes [59, 67].

Some researchers have also suggested that myostatin may activate genes associated with UPS and maximize a proteolytic process [68]. However, experiments did not show the modulation of MuRF-1 and MAFbx by myostatin [68].

On the other hand, myostatin seems to inhibit the activity of important factors associated with muscle protein synthesis and regeneration, such as Akt, satellite cells and myogenic factors (e.g., MyoD) [58, 67, 69, 70]. In fact, McCroskery et al. [70] showed that myostatin knockout mice displayed higher number of satellite cells activated in the cell cycle and in steady state per unit of muscle fibers, together with faster proliferation of myoblast when compared to the controls [70].

Myostatin was increased in some animal and human models of muscle atrophy, for example, food deprivation, muscle disuse, and muscle denervation [68, 69, 71, 72]. Moreover, gene electrotransfer of a myostatin expression vector was found to induce 20% of muscle atrophy in tibialis anterior muscle of rats [58].

It has been suggested that in poststroke patients, atrophy disuse and denervation would account for increased myostatin. Moreover, myostatin is sensitive to glucocorticoids, which are highly consumed by poststroke patients [4]. However, data remain controversial. In animals undergoing transient focal cerebral ischemia, myostatin increases exponentially in both paretic and nonparetic limb 3 days after cerebral occlusion [47, 73]. However, SMAD 2 and SMAD 3 present downregulation [47]. The only study in humans, to our knowledge, was performed by von Walden et al. [57]. They investigated atrophied lower limb muscle in patients who suffered a stroke in the previous 9 years and found that their biopsy presented lower myostatin expression when compared to older healthy adults [57].

Thus, it is possible that myostatin modulates muscle atrophy at the beginning of the phenomenon, but not during its progression. These findings are corroborated by experiments involving nonstroke patients, which demonstrated that myostatin levels decrease few days after the beginning of muscle atrophy, during the peak loss of muscle mass [58, 69].

In view of such a range of candidate mechanisms, it is difficult to pinpoint the precise process responsible for muscle atrophy in poststroke patients, despite the indications provided by the fact that some pathways (i.e., UPS, autophagy, and myostatin) are increased during this process. Furthermore, further studies involving both pathways in animal models of stroke and in humans are needed, mainly to understand the molecular signaling.

**Figure 1:**
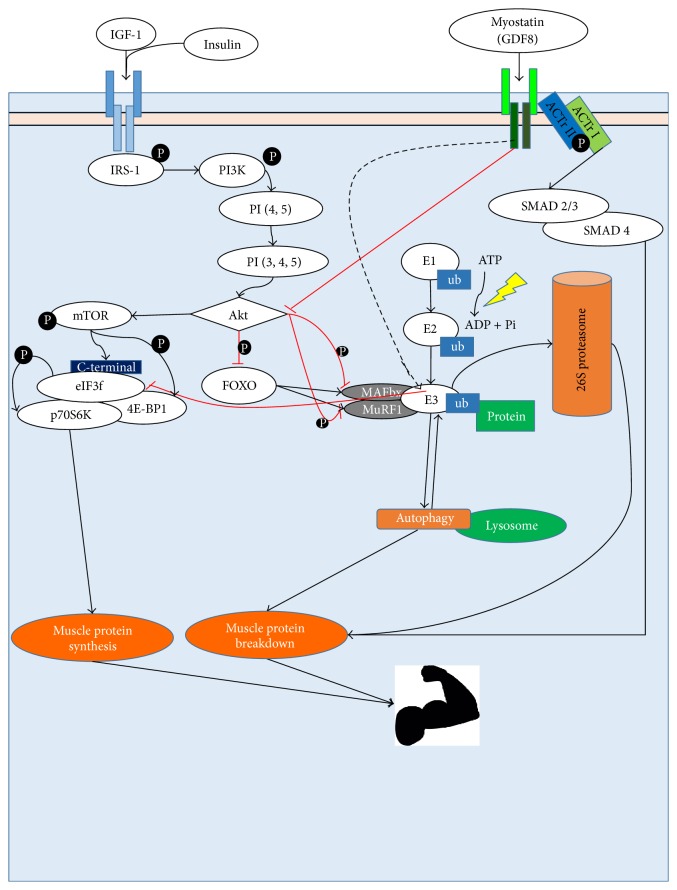
Anabolic and catabolic pathways regulating muscle mass. P = phosphorylation; Ub = ubiquitin.

Figure 1 shows the anabolic and catabolic pathways indicated to regulate muscle mass in poststroke patients.

## 4. Low-Grade Inflammation as a Trigger Factor to Activation of Muscle Atrophy Pathways

Inflammatory process is the mechanism of the immune system in charge of protecting the organic system against harmful agents and restoring homeostasis. Once activated, the components of the immune system return to prestress levels in few days or, at most, weeks. However, this is due to the decreased capacity of the physiological organic system to cope with stressful factors (e.g., reactive oxygen species (ROS)), as may occur due to aging—leading to inflammatory phenotype—or even in response to a pathological state, leading to a chronic low-grade inflammation condition [74, 75].

A number of physiological disorders, such as chronic pulmonary obstructive disease (CPOD), rheumatoid arthritis (RA), cancer, and inflammatory myopathies, also called myositis (i.e., polymyositis, inclusion body myositis, and dermatomyositis), have been found to be associated with elevated proinflammatory markers [8, 9, 76]. Furthermore, inflammation has been suggested to be a common factor leading to increased activity of atrophy-related catabolic pathways, such as UPS and autophagy, during aging (i.e., senescence and senility) and in chronic degenerative states (i.e., cachexia) [8, 9].

Animal studies have demonstrated the regulation of proinflammatory cytokines in myoplasticity. Evidence in the literature has shown that infusion of recombinant tumor necrosis factor-alpha (TNF-*α*) and TNF-*α* plus interleukin type-1 (IL-1), both proinflammatory cytokines, increases muscle catabolism in rats [77]. In mutant mice with overexpression of proinflammatory cytokines (i.e., interleukin type-6 (IL-6)), muscle mass presents exacerbated atrophy when compared to the wild type [78, 79]. However, inhibition of IL-6 receptor prevents muscle wasting and increase in UPS activity [79].

Inflammatory process may be also associated with the muscle atrophy phenotype caused by cachexia syndrome in some pathological conditions, since proinflammatory cytokines expression (i.e., mRNA) and production are increased in animal models of heart failure [80, 81] and cancer cachexia [82].

Regarding human beings, cross-sectional studies have found an association between increased concentrations of proinflammatory cytokines (e.g., IL-6 and TNF-*α*), their soluble receptors, and acute phase proteins (i.e., C-reactive protein (CRP)) and syndromes associated with low muscle mass in elderly [83, 84], cancer cachexia [85], and chronic heart failure [86]. Moreover, although not conclusive, a number of longitudinal studies have suggested that high concentrations of inflammatory markers may indicate a higher degree of muscle atrophy after 3 years [87].

Between the myriad of cytokines which may be associated with muscle atrophy, TNF-*α* (also called cachectin) seems to be the most likely candidate, due the high number of catabolic pathways that its molecule are involved in [88]. In fact, increased TNF-*α* is found in muscle wasting related conditions, such as cancer, heart failure, COPD, and sarcopenia [83–86, 89].

However, in poststroke patients, this scenario remains unclear. Indeed, the expression of mRNA TNF-*α* was increased in the vastus lateralis muscle, contralateral to the lesion, in chronic stroke survivors when compared to ipsilateral limb [90]. Nevertheless, this result was not confirmed in a more recent study [57]. Regarding systemic measurements, an increased level of TNF-*α* in the serum and cerebrospinal fluid was found in patients 24 hours, one week, and two weeks after the stroke. These increases were associated with infarct volume and severity of neurological impairment [91]. Corroborating these clinical findings, TNF-*α* blocking was found to reduce the volume of infarction after occlusion of the cerebral artery in mice [92].

Taken together, these data indicate a possible modulation of TNF-*α* in the skeletal muscle and in the central nervous system (e.g., motor cortex), which in turn affects functionality and, consequently, muscle mass (e.g., hemiparesis, spasticity, rigidity, balance, and coordination changes).

TNF-*α* effects on muscle wasting my be said to be due to inhibition of protein synthesis (because of alterations in the levels of anabolic hormones, such as IGF-1), a result of phosphorylation of IRS-1 and IRS-2 receptors, inhibition of satellite cells activity, and reduction of MyoD expression [88, 93–95]. Moreover, TNF-*α* seems to downregulate the synthesis of myosin heavy chain of slow-twitch fibers and increase their degradation [96, 97], which may account for the changes in fiber phenotype in the muscles of chronic stroke survivors.

Regarding proteolysis, we may hypothesize that the process is mediated by TNF-*α* through direct (e.g., apoptosis) and indirect mechanisms [88]. The indirect mechanisms are based on the capacity to recruit other proinflammatory cytokines and immune system cells, particularly by the stimulus to cause nuclear factor kappa-light-chain-enhancer of NF-*κ*B activation [88, 96, 98]. In this sense, some researchers have suggested that muscle atrophy mediated by TNF-*α*/NF-*κ*B activity may be the most powerful stimulus to muscle atrophy [18, 19].

NF-*κ*B is a molecular signaling pathway originating from an evolutionary process, which plays a critical role in the activity of the immune system, regulating some physiological and pathological process, increasing the levels of inducible nitric oxide (iNOS), with subsequent formation of reactive oxygen species, which results in oxidative damage [99]. Moreover, given its capacity to increase proinflammatory cytokines synthesis, NF-*κ*B is known as the master regulator of inflammatory state [99, 100].

NF-*κ*B may be activated by some stimulus, such as proinflammatory cytokines and ROS [96, 99–102]. However, when not activated, NF-*κ*B remains in the cytoplasm, inhibited by I*κ*B*α* activity, a molecule from I*κ*B kinase (IKK) family [100, 102]. However, due to stimulation, another IKK molecule, I*κ*B*β* (IKK), phosphorylates and activates I*κ*B*α*, leading to its ubiquitination and eventually to the degradation on 26S proteasome [100, 102]. Since I*κ*B*α* acts by inhibiting the activity of NF-*κ*B, degradation makes NF-*κ*B free to translocate to the nucleus and change the gene transcription of proinflammatory cytokines [100, 102]. Regarding TNF-*α*, NF-*κ*B activation by this proinflammatory cytokine is mediated through 1 TNF-*α* receptor (TNFR1) and/or 2 TNF-*α* receptor (TNFR2) [88].

Data from literature points to the capacity of NF-*κ*B to contribute to muscle atrophy, since this molecule is increased in different models of muscle atrophy, that is, disuse and cancer cachexia [100]. Besides, in a seminal study, Cai et al. [100] have developed transgenic mice with NF-*κ*B overexpression and found severe muscle atrophy in these animals, due to a sharp decrease in the cross-sectional area of muscle fibers, when compared to the wild type. Interestingly, this phenotype was followed by increased MuRF-1 and proteasome activity, indicating a possible interaction between NF-*κ*B and UPS. Moreover, when the authors blocked NF-*κ*B, muscle mass was restored [100].

In view of these findings, it is possible to infer that NF-*κ*B activation by NF-*κ*B in poststroke patients may contribute not only to muscle atrophy, but also to endothelial dysfunction, causing a positive feedback, ROS production, and inflammation, while maintaining NF-*κ*B activation. This phenotype has been already suggested for other pathological states, such as diabetes mellitus type II [99]. However, data concerning poststroke patients remain unclear in the literature.

Myosteatosis is another condition found in the paretic limb of poststroke patients. This condition is characterized by fat deposition in the skeletal muscle, as well as by the amount of fat mass around the muscle. Evidence in literature suggests that myosteatosis is increased in the atrophied paretic limb in poststroke patients, when compared to the ipsilateral limb [103, 104]. The differences in intramuscular fat content between contralateral and ipsilateral limb can reach 48% in favor of the impaired limb [20]. This may account for the larger increase in fat mass relative to muscle area in the paretic limb relative to ipsilateral limb [20, 73, 103].

The white adipose tissue (WAT) is not just a deposit of triacylglycerol and, consequently, energy but also an active endocrine organ capable of synthesizing and secreting proinflammatory cytokines [85, 105–109]. Increased expression and protein content of proinflammatory cytokines are found in syndromes related with muscle wasting, as cancer cachexia [85, 105–110].

Indeed, visceral (i.e., mesenteric, epididymal, and retroperitoneal) and subcutaneous WAT in cancer cachexia present increased expression of proinflammatory cytokines (i.e., TNF-*α*, IL-6, and IL-1*β*), acute phase proteins (i.e., C-reactive protein (CRP)), and chemotaxis factors (i.e., monocyte chemoattractant protein-1 (MCP-1)), concomitant to increased NF-*κ*Bp65, IKK-*α*, and toll-like receptor 2 (TLR2) expression [85, 105, 106, 109, 110]. Moreover, cancer patients with cachexia have a lower number of macrophages (M*ϕ*) with an anti-inflammatory phenotype (M2) when compared to weight-stable cancer patients, and this may be associated with fat deposition [110].

However, data showing an association between muscle atrophy and WAT in patients with cachexia should be carefully extrapolated to poststroke patients, since data so far have indicated that proinflammatory phenotype may be tumor-dependent [106, 110]. On the other hand, muscle skeletal remodeling is also observed during aging, and older adults show up 2.5-fold more myosteatosis than young adults [111, 112]. Interestingly, intramuscular fat infiltration is negatively associated with muscle volumes of 15 muscles of the lower limbs in the elderly [112], which points to a strong association between myosteatosis and muscle atrophy. Moreover, intramuscular fat infiltration is associated with increased expression of proinflammatory cytokines (i.e., IL-6) [113].

Thus, we may hypothesize that the increased myosteatosis observed in poststroke patients is associated with muscle atrophy due a low-grade inflammatory state, possibly modulated by NF-*κ*B activity. Moreover, some researchers have found that peroxisome proliferator activated receptor (PPAR) may contribute to myosteatosis, leading to muscle atrophy [114].

PPAR is a member of a nuclear receptor family of ligand-dependent transcriptions factors and comprises 3 PPAR subtypes: PPAR*α* (NR1C1), PPAR*β*/*δ* (NR1C2), and PPAR*γ* (NR1C3), which have wide range of effects in the physiological system [114–116].

The main activity of PPAR is located in the adipose tissue, where this molecule regulates positively (i.e., upregulation) the network of adiposity-specific genes, controlling lipid metabolism (i.e., adipogenesis), adipocyte differentiation (i.e., white and brown), and glucose homeostasis (i.e., insulin sensitivity) [114, 115, 117].

Moreover, recent evidence has suggested that PPAR may have an anti-inflammatory propriety, inhibiting inflammatory mediators, such as cytokines (i.e., IL-6 and TNF-*α*), adhesion molecules (vascular adhesion protein 1 (VCAM1)), acute phase proteins (CRP), IKK, and NF-*κ*B [114–117]. Moreover, PPAR may counterregulate inflammatory conditions induced by pathological states (i.e., obesity and liver fibrosis) [115]. Besides, PPAR may contribute to increasing insulin sensitivity, through adiponectin synthesis [117].

However, inflammatory cytokines, such as TNF-*α*, may inhibit PPAR, leading to a wide range of alterations, contributing to impaired glucose metabolism, hyperinsulinemia, ROS production, and possibly atherosclerosis, along with increased inflammatory state [114]. Thus, decrease in PPAR activity may lead to a catabolic environment associated with muscle atrophy. Nonetheless, the role of NF-*κ*B activity and PPAR*γ* in the muscle atrophy of poststroke patients should be further studied and tested.

Interestingly, the increase in intramuscular fat content in poststroke patients has also been widely suggested as a trigger factor to impaired glucose metabolism, a condition that may reach about 80% of the chronic stroke patients [73, 103, 118]. In fact, increased intramuscular fat infiltration, regardless of visceral fat, contributes to the genesis of dyslipidemia, and impaired insulin sensitivity and glucose uptake, causing hyperinsulinemia and hyperglycemia, thus providing a favorable environment for chronic conditions, such as hypertension and diabetes mellitus type II [103, 104, 118, 119]. Therefore, PPAR maybe the pathway underlying impaired glucose metabolism due to increased intramuscular fat content in poststroke patients.

**Figure 2:**
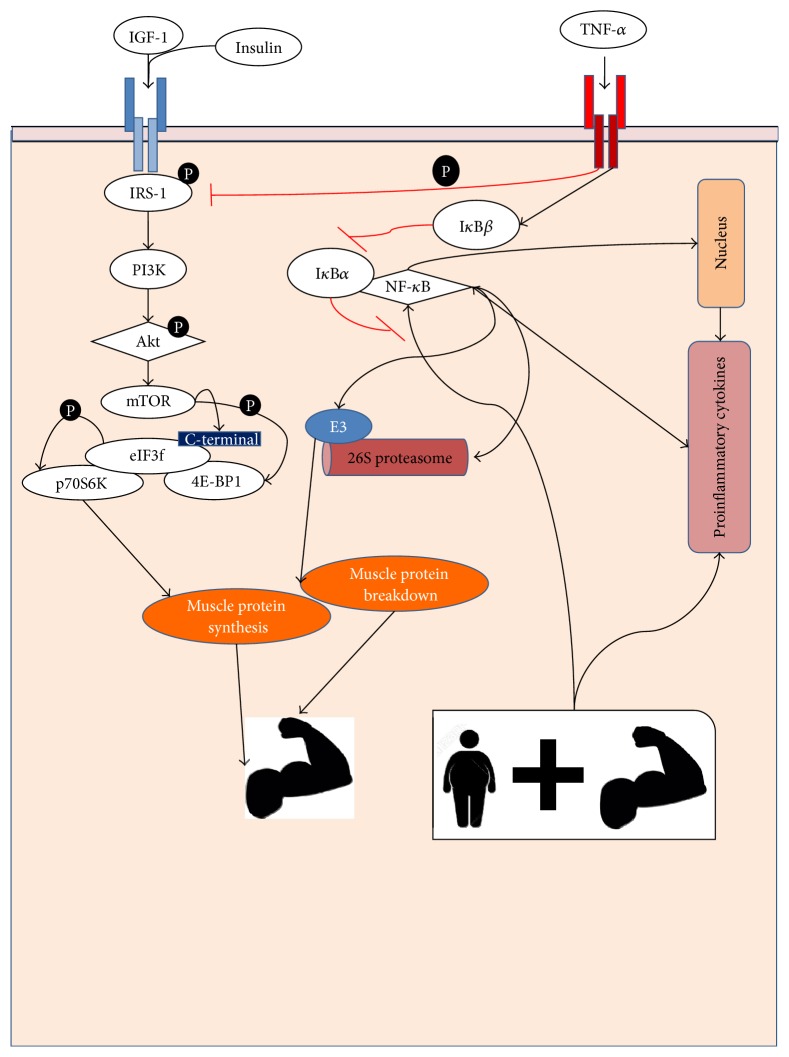
Influence of the inflammatory factors in the regulation of muscle mass.

Figure 2 shows the inflammatory factors indicated to regulate muscle atrophy in poststroke patients.

## 5. Physical Exercise and Stroke

As aforementioned, poststroke patients are generally affected by morphofunctional and cognitive complications, which impairs their capacity to perform the daily life activity and basic and advanced self-care, leading to sedentary behavior and increased hospitalization.

On the other hand, physical exercise (PE) has been postulated by international organizations (i.e., American Heart Association (AHA) and American Stroke Association (ASA)) as a useful tool for the rehabilitation of poststroke patients, since PE may counterregulate the most of the deleterious effects of stroke in the organic systems [10]. Indeed, several reviews have pointed out that PE can elicit improvement in cognition, upper and lower limb motor function, cardiovascular performance, cardiovascular risk factors (e.g., triglycerides), fatigue resistance, balance, gait, and mobility [10, 12, 120].

Most studies on the role of PE in poststroke patients have focused on aerobic exercise. A recent systematic review showed that most clinical trials studying the effect of aerobic physical exercise on stroke patients have used short-term interventions (6–8 weeks), with a mean frequency of 3 sessions per week, and a 30–40-minute exercise duration [120]. Regarding exercise intensity, moderate and progressive (moderate to moderate-intense) intensities prevailed [120].

Increase in cardiorespiratory fitness (15–18%) is the most cited alteration after moderate aerobic exercise, and it occurs even after short-term exercise protocols (i.e., 8 weeks) [10, 120–123]. However, the beneficial effects of moderate aerobic exercise in poststroke patients are not restricted to cardiorespiratory fitness, and studies have demonstrated increase in mobility (e.g., get up and go test (GUG)) and motor function, [123], as well as decrease in cardiovascular risk factors, such as hyperinsulinemia [122].

Changes in the cognitive domain after moderate aerobic exercise have also been the focus of some studies. In the experiment of Quaney et al. [123], the authors did not observe significant changes in selective attention, resistance to interference, working memory, and learning after 8 weeks of moderate aerobic exercise (70% of HRmax), which comprised 45 m sessions. A recent meta-analytic review [124] has challenged those findings, arguing that the practice of physical exercise is able to improve cognition in poststroke patients, even when patients presented depressive symptoms and high anxiety levels [124]. However, these findings should be carefully evaluated since not many studies have focused on cognitive improvements, and these were generally assessed as a secondary outcome. Also, cognitive assessment would require more specific methodological tools.

Some studies have dealt with outcomes that are not generally investigated as clinical outcomes but are nevertheless useful to understanding the phenomenon associated with stroke and the impact of physical exercise on this condition. Ivey et al. [121], for example, have studied the effects of PE on blood flow in poststroke patients. Impaired blood flow is usually associated with endothelium dysfunction, ROS, and inflammation, posing a risk factor to myocardial infarction and recurrent stroke. After 6 months of moderate intensity aerobic exercise (60–70% HR reserve), volunteers showed significant increase in rest blood flow, 25% and 23% in paretic and nonparetic limb and in reactive hyperemia blood flow, 25% and 22% in paretic and nonparetic limb [121].

Some researchers have argued that although aerobic exercises may have beneficial effects on poststroke patients, they would be poorly tolerated in this population [125]. In this context, resistance training exercises have been suggested as an interesting alternative, since they are easier to modulate than aerobic exercise and as such more manageable for poststroke patients [12, 125]. Also, resistance training is able to elicit increase in physical function (i.e., muscle strength and power) and mobility [126, 127]. Nevertheless, resistance training has been poorly studied and the outcomes assessed are generally restricted to physical function. Further studies are required to evaluate other important outcomes, such as improved cognition [125].

Interestingly, few studies aimed to record the effects of PE on muscle mass. Regarding aerobic exercise, Ivey et al. [122] have undertaken one of the few studies investigating this issue, but they did not observe significant alterations. In relation to resistance exercise, Ryan et al. [73] developed the only study that reported the effects of this kind of intervention on muscle mass in poststroke patients. Other researchers have concentrated on the effects of resistance training on myostatin expression. Their findings demonstrated that 12 weeks of resistance exercise until muscle failure was able to elicit an increase in muscle mass of the paretic limb (13%) and nonparetic limb (9%) [73]. A decrease of 49% of myostatin mRNA on paretic limb and 27% on the nonparetic limb was also reported [73].

The rather limited number of studies in the literature dealing with the effects of physical exercise on muscle mass makes it difficult to offer a well-informed assessment of the effects of physical exercise on muscle mass. More studies on this association are required, since muscle atrophy and sarcopenia are linked to hemodynamic [128–130], metabolic [131], and functional alterations [132, 133], which contribute to poor outcomes in poststroke patients. Additionally, increase in muscle mass is associated with increase in physical function and mobility [134–136].

## 6. The Anti-Inflammatory Effects of Physical Exercise and the Role of Myokines

Physical exercise has been indicated as a powerful nonpharmacological therapy to decrease inflammatory markers, ameliorate the anti-inflammatory environment, and, consequently, lower chronic inflammation in several diseases (e.g., chronic pulmonary obstructive disease, atherosclerosis, heart failure, and myocardial infarction) [13–16, 137].

In fact, it has been found that chronic moderate and moderate-to-high intensity physical exercise may elicit a decrease in inflammatory factors (e.g., TNF-*α*, IL-1*β*, IL-6, intercellular adhesion molecule (CAM-1), acute phase proteins (CRP), vascular cell adhesion molecule (VCAM-1), and granulocyte-macrophage colony-stimulating factor (GM-CSF)), in different animal models of diseases, such as heart failure [80], myocardial infarction [138], and cancer cachexia-anorexia [139] in healthy [140] and senescent animals [141], as well as in human patients with moderate to severe chronic heart failure (~24% ejection fraction) [142], overweight and obesity [143, 144], diabetes mellitus [143–150], myopathies (i.e., dermatomyositis and polymyositis), rheumatoid arthritis [148], spinal cord injury [149], and systemic lupus erythematosus [151], and in elderly people: those with chronical conditions [150] and the healthy [152].

These alterations can occur locally, affecting the expression of these factors in the cardiac muscle [138], skeletal muscle [80, 140, 146, 147, 150], and adipose tissue [153], in the central nervous system [139, 141] or systemically [142–145, 151, 152]. Furthermore, PE also seems to be effective in increasing anti-inflammatory markers (e.g., IL-10) [80, 107]. Besides, data from literature indicate that alterations in inflammatory factors may also be associated with improved physiological function (e.g., ventricular function; aerobic capacity; insulin resistance) [138, 142, 143, 154].

There are no evidences about the effects of chronic physical exercise on the inflammatory markers in stroke patients. Besides being associated with muscle atrophy and poor prognosis, high inflammatory markers have been linked to elevated risk of recurrent ischemic stroke and cardiovascular events even after adjustment for age, sex, race, comorbidities, and statin use [155].

Several mechanisms may be associated with the anti-inflammatory effects of physical exercise: decrease in the expression of toll-like receptors on monocytes and macrophages, inhibition of the infiltration of immune cells on adipose tissue, changes in the phenotype of macrophages on adipose tissue, and decrease in adipose tissue [13].

For many years, the skeletal muscle was predominantly known by its capacity to generate strength, power, and, consequently, physical movement. Later, researchers hypothesized that some or a single humoral factor would be secreted by the active skeletal muscle and would act by altering the signalization of different molecular pathways [14, 137]. As knowledge about the activity and the properties of these molecules was scarce, they were initially called “exercise factor,” “work stimulus,” and “work factor” [14, 137].

However, accumulated evidence now views the skeletal muscle acting as an active endocrine organ, since the contraction of skeletal muscle in response to a determined load, as observed during physical exercise, may elicit synthesis and release of peptides, hormone-like factors and cytokines—pro- and anti-inflammatory—which, in turn, alter the functioning of tissues and organs [13–15, 137, 156]. Once aggregated, these molecules are termed myokines, and they have been found to be responsible for the interaction between the skeletal muscle and the organic system, due to their action in a paracrine and endocrine and, possibly, autocrine fashion [14, 156].

Regarding the anti-inflammatory effects of the myokines, interleukin-6 (IL-6) is one of the most well-known and studied, being the first to be denominated as a myokine [13, 15, 137, 157]. Besides, IL-6 is considered one of the key myokines which provide the anti-inflammatory effects of physical exercise [13, 15, 137].

Several studies in human beings have demonstrated an increase by over 100-fold in IL-6 levels, as well as mRNA expression, during and after PE, independently of exercise-induced muscle damage and inflammation [158–160]. Moreover, IL-6 is not just an anti-inflammatory cytokine, but evidences indicate its action on glucose metabolism and bioavailability, contributing to beta-oxidation and glucose uptake [160, 161].

In relation to physical exercise, IL-6 seems to be sensitive to alterations on the variables of PE, since running intensity and volume are positively associated with IL-6 levels on plasma [159, 160, 162]. In humans, moderate aerobic physical exercise (75% VO_2max_) and rhIL-6 infusion are able to increase IL-6 levels in blood plasma and, concomitantly, attenuate the increase in TNF-*α* levels after endotoxin infusion [163]. Also, IL-6 plasma levels decreased after cessation of PE or rhIL-6 infusion [158, 159, 162]. Interestingly, cessation of low-levels of rhIL-6 infusion was accompanied by a decrease in anti-inflammatory cytokines: IL-1 receptor antagonist (IL-1ra) and IL-10 [159]. On the other hand, decreased IL-6 levels were found 1,5 h after the end of a strenuous physical exercise, showing a sharp increase (>100x) after the end of exercise session. This was accompanied by IL-1ra values 45-fold higher than preexercise levels, which, in turn, were positively correlated with IL-6 levels [162].

Therefore, these data indicate that the anti-inflammatory effect of physical exercise can be mediated by myokines, mainly IL-6. However, there is not a consensus on whether IL-6 acts directly or through other anti-inflammatory cytokines (IL-1ra, IL-10). Both theories are plausible, and the fact remains that myokines do contribute to an anti-inflammatory environment. Additionally, other myokines, IL-8, IL-15, and the brain derived neurotrophic factor (BDNF), have been linked to angiogenesis, metabolism, neurogenesis, and memory formation [14, 137, 161]. But their effect on inflammatory state have yet to be elucidated.

Therefore, these data indicate that the anti-inflammatory effect of physical exercise may be mediated by myokines, mainly IL-6. However, these myokines have not been studied in a stroke context and inferences are therefore limited. Nevertheless, increase in myokines and decrease in the inflammatory milieu of poststroke patients after physical exercise may improve the prognosis of this population, due to a better physiological environment, one which increases muscle mass, strength, power, and mobility while decreasing cardiovascular risk factors.

**Figure 3:**
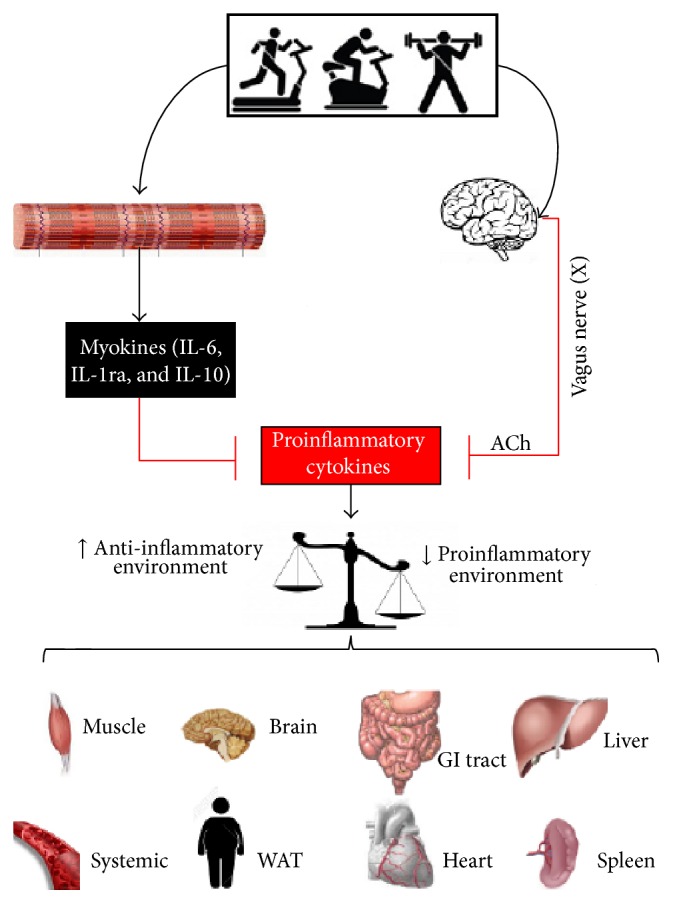
Possible anti-inflammatory pathways modulated by physical exercise. Ach = acetylcholine; *α*7nAChR = receptor *α7* subunit; WAT = white adipose tissue.

Figure 3 shows a schematic representation of the myokines activation in response to physical exercise and its inhibitory anti-inflammatory activity on the inflammatory environment.

## 7. Cholinergic Anti-Inflammatory Pathway

Findings from studies conducted by Tracey group [164–166], as well as from other groups [138, 167, 168], have suggested that the central nervous system (CNS) may act by regulating the central and peripheral inflammatory process [164–166]. This phenomenon occurs due to the activity of the two functional divisions of the autonomous (also called visceral) nervous system—sympathetic (SNS) and parasympathetic nervous system (PNS)—so that each would regulate in a different fashion the immune system and, consequently, the inflammatory process [165, 166, 169].

SNC is known to be activated during “flight or fight” conditions, since it offers the organic system a large blood supply (i.e., increased heart rate and blood pressure), energy substrate (i.e., increased lipolysis), oxygen supply (i.e., bronchial dilation), visual acuity, adrenalin and noradrenalin concentrations, and muscle strength (which could help with survival during Paleolithic and Neolithic period [169].) SNC is increased during situations of allostasis (e.g., hypotension and electrolyte imbalance in order to reestablish the normal functioning). Moreover, this system also acts during dynamic homeostasis, helping to control several physiological systems (e.g., gastrointestinal, cardiovascular, and endocrinal) [165, 166].

Catecholamines (adrenalin and noradrenalin) are synthesized and secreted by the adrenal medulla in the adrenal gland in response to sympathetic activity. Their role in immune cells lies in the fact that they alter their functioning by the modulation of cytokine release, since these cells have *α* and *β* receptors to catecholamines [165].

In turn, PNS acts in an anti-inflammatory fashion, a phenomenon denominated cholinergic anti-inflammatory pathway (CAIP) [164–166, 169–173]. The vagus nerve is an inherent component of this pathway. This nerve is the decimal (X) nerve of the SNC and has afferent, motor, and efferent projections [174]. Regarding afferent projections, these conduct information from sensitive periphery receptors, as chemoreceptors, baroreceptors, and visceral receptors on the thorax and abdomen to the CNS [174].

Concerning CAIP, the afferent vagus nerve fibers act as a peripheral sensory component of the PNS and identify the increase in proinflammatory cytokines from the inflammation, linking the CNS to the immune system [164–166, 169–173].

In the brain, the vagus nerve is found in the nodose ganglion and inside the dorsal vagal complex of the medulla oblongata, which is formed by the nucleus of the solitary tract (NST), dorsal motor nucleus of the vagus, and the area postrema [174]. From there, a neurohumoral and a cholinergic pathway can occur and counterbalance inflammation [174]. The neurohumoral pathway is activated due to the synapse of the NTS with the paraventricular nucleus, a hypothalamic nucleus, which stimulates the synthesis and release of the corticotropin releasing hormone (CRH) [174]. However, the functioning of this pathway remains poorly understood.

On the other hand, projections from the NTS form the efferent arc of the inflammatory reflex, which would act through efferent vagus nerve fibers and neurotransmitters [169]. In summary, CAIP occurs after the inflammatory signalization on the afferent vagus nerve fibers to the NTS; a reflex response mediated by efferent vagus nerve fibers will propagate and culminate in acetylcholine (i.e., ACh), the mainly parasympathetic neurotransmitter, release [165, 166, 169].

Several studies have demonstrated the protective effect of CAIP stimulation, as well as the need of the efferent vagus nerve, on animal models of systemic inflammation [164, 168, 175–177]. In fact, results from experimental studies have found that intravenous (IV) and intracerebroventricular (ICV) pretreatment with CNI-1493 (a pharmacological stimulator of the vagus nerve), carbachol (a cholinergic agonist), pyridostigmine (a peripheral cholinesterase inhibitor agent), and electrical stimulation protected rats against inflammatory factors associated with acute hypovolemic hemorrhagic shock, endotoxin-induced shock, myocardial infarction, and carrageenan-induced acute inflammation, such as increase in serum TNF-*α* levels, edema, neutrophil aggregation, macrophage infiltration, NF-*κ*B protein levels, and loss of IkBa [164, 168, 175–177]. Also, these alterations on inflammatory markers may be associated with increased M2 macrophages, which are characterized by producing mainly anti-inflammatory cytokines and regulatory T cells (Tregs), known by their immunosuppressive capacity [168].

On the other hand, surgical and chemical (i.e., atrophin, an antagonist of cholinergic pathway) vagotomy eliminated the protective effects of chemical and electrical vagus nerve stimulation on inflammatory markers [164]. Furthermore, after surgical vagotomy, the researchers performed new electrical stimulation, but this time on the distal end of the transected right vagus nerve and found an attenuation of the acute inflammation [176].

The activity of CAIP seems to be mediated by ACh, which is the most important neurotransmitter, being the main neurotransmitter release in postganglionic efferent vagal neurons [165, 166, 172]. After the activation of central muscarinic receptor M1 and/or inhibition of the negative regulator of the ACh release (the M2 receptor), ACh is released and acts by using two G protein-coupled receptors, muscarinic and nicotinic [169, 170].

Classically, the most well-known activity of ACh in the organic system is mediating muscarinic receptors, which are found in the hearth and neurons and in the smooth muscle, for example. However, regarding the immunological activity of the ACh, the muscarinic receptor did not seem to have a role in this response. A specific unit of nicotinic receptors, nicotinic acetylcholine receptor *α*7-subunit (*α*7nAChR), is found in immune cells and, once activated, it inhibits cytokine secretion [178, 179]. In fact, Wang et al. [179] have demonstrated that *α*7nAChR is essential to the effectiveness of the anti-inflammatory effect of the cholinergic efferent arc on TNF-*α*, IL-1*β*, and IL-6 concentration in endotoxemic rats, since *α*7nAChR-deficient mice present higher TNF-*α*, IL-*β*, and IL-6 concentrations when compared to the wild type [179].

Furthermore, increased ACh levels, due to activation and inhibition of M1 and M2 muscarinic receptors, respectively, and consequently increase in vagus nerve activity (as demonstrated by Pavlov et al. [170]) will cause inhibitory effects on inflammatory markers (i.e., TNF-*α*, IL-1B, IL-6, and IL-18) through a posttranscriptional mechanism, since ACh did not alter proinflammatory cytokines mRNA in LPS-stimulated macrophage [170, 176].

In relation to stroke patients, evidences in the literature have been indicating that poststroke patients present a phenotype of dysautonomia, mainly characterized by decreased on vagal tonus [11, 180, 181]. Indeed, cross-sectional studies showed impairment of the autonomic control, diagnosed by decreased parasympathetic activity in time domain measures (i.e., root mean square of successive differences (RMSSD) and standard deviation of the normal-to-normal R-R intervals (SDNN)) and frequency domains (high frequency (HF)) of HRV—in right-sided and left-sided ischemic stroke patients when compared to aged-matched healthy control [11, 180, 181]. Also, these results are more evident in patients with right insular involvement, probably due to the association with cardiac control [180].

Besides its negative impact on cardiovascular complications (e.g., increase in ventricular and supraventricular arrhythmias) [11, 180, 181], dysautonomia may be one of the mechanisms responsible for the aforementioned increased inflammatory markers observed locally and systematically in poststroke patients, due to impairment in the CAIP functioning.

Interestingly, CAIP was thought at first to act only as an arc reflex, controlling acutely the inflammatory state and preventing cellular damage [169, 173]. However, some researchers have suggested that CAIP may also act in a chronic fashion, in the pharmacological treatment, and it may be responsible for the anti-inflammatory effects of physical exercise, for example [154, 182]. However, only few experiments have tested this hypothesis [138, 183].

In the research undertaken by Conti et al. [183], the authors tested the hypothesis that physical exercise would positively modulate the deleterious effects of menopause, mimicked through ovariectomy, on autonomic nervous system and inflammatory profile in metabolic syndrome (i.e., SHR more fructose diet) female rats. Animal underwent 8 weeks of low-to moderate intensity combined physical exercise, aerobic (60% maximal running speed) plus resistance training (60% of the maximum load), which was performed 5 days per week. Results showed that combined physical exercise was able to restore dysautonomia and TNF-*α* levels on cardiac tissue. Furthermore, oxidative stress, one of the mechanisms associated with NF-*κ*B activation, was decreased in both cardiac and renal tissue of trained metabolic syndrome female rats when compared to sedentary metabolic syndrome rats [183].

Rodrigues et al. [138] have studied the effect of PE on autonomic control and the potential impact on inflammatory markers in infarcted rats. In this study, infarcted rats underwent a moderate intensity (50–70 of the VO_2max_) exercise program, 1 h per day, 5 days per week, during 3 months. Researchers observed increased HF of the pulse interval, which characterizes parasympathetic activity, in trained group when compared to the sedentary infarcted control group. Moreover, TNF-*α* concentration and TNF-*α*/IL-10 ratio were lower in trained infarcted rats than in sedentary control. Interestingly, one group of rats trained for only two months, followed by a 1-month detraining period. Results of this group were similar to the trained group, presenting increased parasympathetic activity and lower inflammatory profile when compared to the infarcted group. Also, in the same study, the researchers found that CAIP was a plausible candidate mechanism associated with decreased inflammatory profile, since HF was negatively correlated with IL-6 and TNF-*α* concentration on the left ventricle [138].

Unfortunately, the effects of physical exercise on cardiovascular autonomic control and their impact on tissue and systemic inflammation profile remain poorly understood and further studies focusing on this issue are needed. However, recent studies have been demonstrated that direct cortical vagus nerve stimulation (Cvns) and indirect (noninvasive transcutaneous vagus nerve stimulation (nVNS)) vagal nerve stimulation lead to decreased infarct volume (up to 33%), neurological damage, and increased grip strength in rats undergoing acute cerebral ischemia [184, 185]. Furthermore, experiments have shown that the middle cerebral artery occlusion (MCAO) animal model undergoing vagal nerve stimulation presented lower Iba1 and CD68 (microglial markers indicating immune response of brain tissue) and TNF-*α*, IL-6, and IL-1*β* concentrations than sham-control [184, 185].

Taken together, data on the anti-inflammatory effect of physical exercise on the inflammatory markers in animal models of myocardial infarction and menopause and the effectiveness of electrical stimulation of vagus nerves in decreasing inflammation in MCAO animals, make it possible to infer that physical exercise may activate CAIP in poststroke patients and, consequently, contribute to a decrease in inflammatory markers, allowing recovery of the organic system.

Figure 3 shows a schematic representation of the CAIP activation in response to physical exercise, and its inhibitory anti-inflammatory activity on the inflammatory environment.

## 8. Conclusion

We presented several findings from a range of studies which may indicate that proinflammatory markers (e.g., TNF-*α* and IL-6) may be responsible for the activation of pathways associated with muscle atrophy in poststroke patients (e.g., UPS system). On the other hand, physical exercise seems to be a powerful tool to counterbalancing these phenomena, due its capacity to elicit a decrease in inflammatory markers in different animal models of disease (e.g., cancer, myocardial infarction, and heart failure), as well as in human beings. These beneficial outcomes may take place because physical exercises seem to act in an anti-inflammatory fashion, through myokines and the cholinergic anti-inflammatory pathway. Even if these data are mostly inference-based, they may point to the possible mechanism to be further studied and encourage research on inflammation and muscle atrophy and on the effect of physical exercise in poststroke patients.

## Abbreviations

TNFR1: 1 TNF-*α* receptor
TNFR2: 2 TNF-*α* receptor
ACh: Acetylcholine
ADL: Activities of daily live
AHA: American Heart Association
ASA: American stroke association
CAIP: Cholinergic anti-inflammatory pathway
CPOD: Chronic pulmonary obstructive disease
Cvns: Cortical vagus nerve stimulation
CRP: C-reactive protein
GUG: Get up and go test
GM-CSF: Granulocyte-macrophage colony-stimulating factor
GDF8: Growth differentiation factor 8
HF: High frequency
IL-1ra: IL-1 receptor antagonist
iNOS: Inducible nitric oxide
IRS-1: Insulin receptor substrate
IGF-1: Insulin-like growth factor 1
IL-6: Interleukin type-6
ICV: Intracerebroventricular
IV: Intravenous
KO: Knockout
M*ϕ*: Macrophages
mTOR: Mammalian target of rapamycin
MCAO: Middle cerebral artery occlusion
MCP-1: Monocyte chemoattractant protein-1
MAFbx: Muscle atrophy F-box
MuRF1: Muscle ringer finger 1
nVNS: Noninvasive transcutaneous vagus nerve stimulation
NF-*κ*B: Nuclear factor kappa-light-chain-enhancer of activated B
PNS: Parasympathetic nervous system
PI3K: Phosphatidylinositol 3-kinase
ROS: Reactive oxygen species
Tregs: Regulatory T cells
RA: Rheumatoid arthritis
RMSSD: Root mean square of successive differences
6MWT: Six-minute walk test
SDNN: Standard deviation of the normal-to-normal R-R intervals
SNS: Sympathetic nervous system
TLR2: Toll-like receptor 2
TGF-*β*: Transforming growth factor-*β*
TNF-*α*: Tumor necrosis factor-alpha
UPS: Ubiquitin proteasome system
VCAM1: Vascular adhesion protein 1
WAT: White adipose tissue.
